# Beyond Amitriptyline: A Pediatric and Adolescent Oriented Narrative Review of the Analgesic Properties of Psychotropic Medications for the Treatment of Complex Pain and Headache Disorders

**DOI:** 10.3390/children7120268

**Published:** 2020-12-02

**Authors:** Robert Blake Windsor, Michael Sierra, Megan Zappitelli, Maria McDaniel

**Affiliations:** 1 Division of Pediatric Pain Medicine, Department of Pediatrics, Prisma Health, Greenville, SC 29607, USA; maria.mcdaniel@prismahealth.org; 2 School of Medicine Greenville, University of South Carolina, Greenville, SC 29607, USA; michael.sierra@prismahealth.org (M.S.); megan.zappitelli@prismahealth.org (M.Z.); 3 Division of Child and Adolescent Psychiatry, Department of Psychiatry, Prisma Health, Greenville, SC 29607, USA

**Keywords:** pediatric pain, pediatric headache, child psychiatry, pain psychiatry, psychotropics, analgesics, complex pain, SNRI, depression, anxiety

## Abstract

Children and adolescents with recurrent or chronic pain and headache are a complex and heterogenous population. Patients are best served by multi-specialty, multidisciplinary teams to assess and create tailored, individualized pain treatment and rehabilitation plans. Due to the complex nature of pain, generalizing pharmacologic treatment recommendations in children with recurrent or chronic pains is challenging. This is particularly true of complicated patients with co-existing painful and psychiatric conditions. There is an unfortunate dearth of evidence to support many pharmacologic therapies to treat children with chronic pain and headache. This narrative review hopes to supplement the available treatment options for this complex population by reviewing the pediatric and adult literature for analgesic properties of medications that also have psychiatric indication. The medications reviewed belong to medication classes typically described as antidepressants, alpha 2 delta ligands, mood stabilizers, anti-psychotics, anti-sympathetic agents, and stimulants.

## 1. Introduction

Children and adolescents with recurrent or chronic pain and headache are a complex and heterogenous population. Pain diagnoses are evolving and sometimes controversial. Within this population, co-morbidities are frequent, and patients often exist on spectrums of central sensitization and active disease [1]. Central sensitization refers to the process where proposed spinal and supraspinal mechanisms alter and amplify pain to a degree that is potentially disconnected from the degree of tissue injury (i.e., nociplasticity). It is distinct from neuropathic pain in that there is no identified or suspected injury to the peripheral or central somatosensory system. Central sensitization is not mutually exclusive of active, ongoing disease, and this construct can be seen in changes to the 11th version of the International Classification of Disease (ICD-11) where pain can be classified as primary or secondary in nature with many patients having both [2]. For instance, patients with inflammatory bowel disease may be in fluctuating states of active or remitted disease that may or may not fully correlate with their experience of pain, and this may be explained by varying degrees of sensitization of the nervous system. The patient may have pain secondary to inflammation from Ulcerative Colitis, and primary pain arising from visceral hyperalgesia (i.e., Irritable Bowel Syndrome) that can heighten the sensation of pain during periods of gastrointestinal inflammation. This can also lead to pain in the absence of active inflammation during periods of remission. In other words, the patient may alternatingly have pain as a symptom of a disease (i.e., Ulcerative Colitis) and have pain as the primary disease process (i.e., Irritable Bowel Syndrome). 

Independent of the co-existence of active disease and central sensitization, children and adolescents with chronic pain have increased rates of mental health and developmental disorders compared to their peers without pain [3]. The etiology of this co-morbidity is an active and rich area of ongoing research that is beyond the scope of this review, but it should be stated explicitly that chronic pain and headache in children is a complex biopsychosocial phenomenon and not a mental health disorder. Investigation for occult primary mental health and developmental disorders often expands opportunities for evidence-based treatment and identifies contributing factors that can be addressed, but treatment of those co-morbidities is frequently insufficient to alleviate pain by itself. 

Due to the complex nature of pain, generalizing pharmacologic treatment recommendations in children with chronic pain is challenging. This is particularly true of the complicated patients with co-existing painful and psychiatric conditions. There is an unfortunate dearth of evidence to support many pharmacologic therapies to treat children with chronic pain and headache [4]. Multiple studies also demonstrate similarly high placebo response rates in children and adolescent for chronic pain, migraine, and psychiatric conditions when compared to adults [5,6]. Accurately identifying co-morbid contributions to chronic pain and headache, including occult developmental and psychiatric disorders, such as Attention-Deficit-Hyperactivity Disorder (ADHD) and Post-Traumatic Stress Disorder (PTSD), likely enhances the odds of improving pain [7,8]. Patients are best served by multi-specialty, multidisciplinary teams to assess and create tailored, individualized pain treatment and rehabilitation plans. As with many things in medicine, appropriate patient selection may be critical to achieve therapeutic success.

This literature review hopes to supplement the available treatment options for this complex population by reviewing the pediatric and adult literature for analgesic properties of medications with common use in child psychiatric practice.

## 2. Methods

As mentioned previously, the treatment of complex pain and headache is a wide-ranging field that spans multiple specialties and disciplines. Within pediatrics, this is further complicated by the need to extrapolate data from adults with pain conditions that are rarely seen in youths, such as painful diabetic peripheral neuropathy. It is well-known that complex pain and headache are highly comorbid with psychiatric conditions. Therefore, this review aims to highlight specific medications and medications classes that are used within the field of child psychiatry that may have evidence of analgesic properties. To orient the non-psychiatrist, the role of these agents in treating pediatric psychiatric conditions will be reviewed and discussed. This review will also describe their mechanisms of action, notable side effects, and evidence-base (or lack thereof) supporting their use for specific pain conditions. The review will state whether the evidence was extrapolated from adult populations or was gathered from pediatric populations. This is not an exhaustive list of all the medications used in child psychiatry and/or pain. Based on experience treating patients in a multi-specialty manner, the authors chose to review select medication classes that seemed the most relevant this heterogenous population. These agents consist of anti-depressants, alpha 2-delta ligands (i.e., gabapentinoids), mood stabilizers, atypical antipsychotics, and anti-sympathetic agents. We recognize that complex pain and headache may require long-term medication use and the authors have excluded habit-forming (i.e., opiates and benzodiazepines) or newly emerging controversial medications (i.e., cannabinoids).

This review may be of use to the practicing clinician treating patients with complex co-morbidities. Special attention is given to adolescent headache. This is not because it is always complex or chronic, but because it is prevalent and often disabling. It also frequently co-exists in patients who have multiple areas of pain and is a common side effect of many medications. Careful attention to the presence of headache impacting daily function may guide pharmacotherapy to an appropriate regimen that limits polypharmacy and promotes therapeutic success. To review the literature, PubMed and the Cochrane Library databases were reviewed for high-level adult analgesic and psychiatry evidence, as well as all available pediatric literature demonstrating analgesic potential of these agents to blend the literature of these specialties.

## 3. Antidepressants

Emerging data suggest that the pathogenesis of both depression and chronic pain may involve inflammatory processes [9,10]. This may partly explain the increased co-prevalence of depression in patients with chronic pain. Inflammation in both scenarios can lead to heightened arousal and subsequent destabilization of the hypothalamic-pituitary-adrenal axis and dysregulate the autonomic nervous system. The immune system is also affected, leading to the overproduction of inflammatory cytokines. Despite the link between pain and depression, the analgesic effects of antidepressants appear to be independent from their effects on mood [11]. The analgesic effect of antidepressants may be mediated by the serotonergic and noradrenergic effects of the descending pain-modulatory system that is coordinated by the periaqueductal gray in the midbrain [12]. Antidepressant effects on pain may manifest within a week, whereas effects on depression can take up to 12 weeks [13]. Selective Serotonin Reuptake Inhibitors (SSRIs), Serotonin and Norepinephrine Reuptake Inhibitors (SNRIs), mirtazapine and bupropion seem to have similar dose requirements to treat pain as depression, but Tricyclic Antidepressants (TCAs) used in adults usually require lower doses for pain [14]. Antidepressants with mixed receptor or noradrenergic mechanisms appear to have the most efficacy in adults due to activity on the descending pain-modulatory system [15,16]. Therefore, SSRIs provide significantly less analgesia than TCAs and SNRIs, and probably less than other novel antidepressants such as mirtazapine and bupropion. The following section will discuss the role of antidepressants in treating pain in more detail.

### 3.1. Selective Serotonin Reuptake Inhibitors

Selective serotonin reuptake inhibitors (SSRIs) are the most prescribed antidepressants in child and adolescent psychiatry [17]. They are relatively safe and cause fewer side effects than other antidepressants. Medications in this class increase serotonergic transmission by blocking the serotonin reuptake transporter. Select agents have approval from the U.S. Food and Drug Administration (FDA) to treat major depression and obsessive-compulsive disorder in children and adolescents [18]. SSRIs are not a homogenous group and the evidence supporting their use in pediatric mental health disorders does not generalize across the entire class. FDA studies in children are limited and therefore FDA-approval for SSRI use in children does not reflect the standard of care within pediatric psychiatry. For instance, only fluoxetine and escitalopram have FDA-approval for the treatment of pediatric major depression, but sertraline and citalopram are often prescribed, especially when on-label treatments fail. Moreover, no SSRIs are FDA-approved to treat anxiety disorders in children and adolescents, but there is evidence that multiple SSRIs reduce social anxiety, separation anxiety, and generalized anxiety disorder [19]. Overall, adolescents respond better to SSRIs than children, and depressive symptoms are ameliorated at low-to-medium doses whereas anxiety and Obsessive-Compulsive Disorder (OCD) symptoms tend to require medium-to-high doses. Fluoxetine is often the first SSRI trialed in youths due to it having the most studies supporting its benefit and a favorable safety profile in pediatric patients [20].

With respect to analgesic mechanisms, most SSRIs are similarly inert except for fluoxetine which has weak sodium channel inhibition and anti-prostaglandin activity [16]. The available evidence suggests that SSRIs have mild analgesic benefits in some adult patients, but that it is generally not a front-line therapy [16,21,22]. SSRIs have not been studied for pediatric and adolescent chronic pain outside of the study of citalopram to treat pediatric functional abdominal pain. The improvements in functional abdominal pain may be related to improvements in the gut–brain axis. Two randomized control trials (RCTs) demonstrated benefit above placebo at doses of 20–40 mg/day [17]. One of the major advantages of SSRI therapy is to provide central modulation without constipation or other anticholinergic side effects. In fact, SSRIs tend to generate pro-motility effects that may benefit patients with chronic constipation. It is important to note that citalopram has a warning on doses > 40 mg/day due to significant QTc prolongation, and all SSRIs carry an FDA black box warning that some children, adolescents, and young adults with major depression or other psychiatric disorders may experience an increase in suicidal thoughts.

### 3.2. Tricyclic Antidepressants

Tricyclic antidepressants are multi-mechanistic agents with good evidence supporting their analgesic efficacy for various neuropathic and wide-spread pains, as a migraine prophylactic in adults, and for chronic tension-type headache [14,15,23,24]. As a class, TCAs possess serotonergic and noradrenergic reuptake inhibition, N-Methyl-D-aspartic acid (NMDA) antagonism, sodium and calcium channel inhibition, kappa and delta opioid receptor agonism, and anti-inflammatory activity via prostaglandin and tumor necrosis factor (TNF)-alpha modulation [16]. The anti-inflammatory effects of TCAs may contribute to benefits seen in some pre-clinical models; however, given the multiple mechanisms of action, it is unclear if analgesic activity is mediated by similar pathways in all conditions [25,26,27,28]. The predominance of clinical studies took place over twenty years ago with few updated studies examining the effects of amitriptyline in patients with inflammatory arthritis and evidence of central sensitization. Nonetheless, TCAs appear to have a role as a first-line pharmacotherapy for the treatment of various adult chronic pain syndromes either as monotherapy or in combination with other agents [14,29,30]. There is also evidence in adults to support use of amitriptyline for functional gastrointestinal (GI) disorders, chronic low back pain, and neuropathic pain [13,31,32]. Typical dosing in adults ranges from 25–75 mg at bedtime; however, studies for the treatment of adult fibromyalgia suggest a lack of additional benefit for doses above 25–30 mg per day [33].

Despite frequent use in pediatrics, the evidence supporting amitriptyline use is not very strong [4,34]. In pediatric functional abdominal pain, amitriptyline has shown improved quality of life, reduced right lower quadrant pain (but not other locations of abdominal pain), and reduced anxiety [17]. Patients with mild-moderate pain responded better than those with severe pain. Like other pediatric studies, large placebo effects were seen across these studies. Similarly, several recent analyses cast doubt on the efficacy of amitriptyline for adolescent episodic migraine. The *Childhood and Adolescent Migraine Prevention* (CHAMP) trial performed a multi-center, randomized, double-blind, placebo-controlled crossover study of amitriptyline, topiramate, and placebo and found no evidence of benefit over placebo [35]. Later network meta-analysis supported this finding [36]. Amitriptyline may continue to have a role in preventing adolescent migraine when used in combination with Cognitive Behavioral Therapy (CBT), but increasing evidence suggests that its benefits as a monotherapy are not superior to placebo [37].

TCAs are notable for being anti-cholinergic, anti-alpha-1 adrenergic, and anti-histaminic that can be counterproductive in cases of chronic constipation, orthostatic dizziness, and obesity. They are metabolized by CYP2D6 and prone to risks from hyper-metabolizers and under-metabolizers, including QTc prolongation. They are also prone to interactions with CYP2D6 inhibitors, most notably fluoxetine, bupropion, cannabidiol, sertraline, and duloxetine, which can all increase amitriptyline levels and contribute to adverse effects.

### 3.3. Serotonin and Norepinephrine Reuptake Inhibitors

Serotonin and norepinephrine reuptake inhibitors (SNRIs) differ from SSRIs in that SNRIs increase both serotonergic and noradrenergic neurotransmission. This mechanism of action suggests SNRIs may be effective in psychiatric patients who fail to respond to SSRIs, particularly those with higher rates of fatigue and psychomotor slowing [20,38]. SNRIs are multi-mechanistic, similar to TCAs, but they achieve this without significant affinity for muscarinic, histaminergic, or alpha-1 adrenergic receptors and therefore cause fewer side effects by comparison [13,16]. SNRIs have notable within-class variations, but generally inhibit the reuptake of serotonin at lower doses and norepinephrine at higher doses to varying degrees. For instance, at lower doses, the side effects of duloxetine and venlafaxine are similar to SSRIs (e.g., nausea, headache) while at higher doses they tend to include insomnia, activation, dry mouth, and hypertension that are more characteristic of noradrenergic activity [20]. SNRIs share the black box warning for risk of suicidality in children, adolescents, and young adults seen with SSRIs. Commonly used medications include duloxetine, venlafaxine, and milnacipran. Newer agents such as desvenlafaxine and levomilnacipran have not been well studied for pain.

#### 3.3.1. Duloxetine

Duloxetine has a 10-fold affinity for 5-hydroxytryptamine (5-HT) over norepinephrine (NE) receptors [38]. It has FDA indications for treatment of major depression, generalized anxiety, neuropathic pain, musculoskeletal pain (particularly chronic low back pain), and fibromyalgia in adults, but is only approved for generalized anxiety and juvenile fibromyalgia [39]. Of the SNRIs, duloxetine has the most evidence to support its use to treat chronic pain syndromes in adults. It also demonstrated consistent analgesia in chemotherapy-induced polyneuropathy (CPN) [40]. Duloxetine was shown to be superior to venlafaxine for CPN, and the proposed mechanisms may not only include class-mediated central noradrenergic activity, but also a duloxetine-specific effect reducing intracellular inflammatory messengers including the mitogen-activated protein kinase (MAPK) and nuclear factor kappa-light-chain-enhancer of activated B cells (NF-kB) pathways that may underlie platinum-induced neural toxicity [40,41].

Generally, the analgesic activity of duloxetine does not occur until 60 mg per day in adults [16]. Clear analgesic dose–response curves are not available, though doses of 60–120 mg have been shown to be effective in various studies. In the *Combination vs Monotherapy of pregabalin and duloxetine in Diabetic Neuropathy* (COMBO-DN) study, combination therapy of 60 mg of duloxetine with pregabalin was shown to be only slightly superior to high-dose duloxetine alone (120 mg), suggestive of at least some dose–response benefits with higher doses. However, given the higher rates of noradrenergic side effects with increasing duloxetine doses, the risk-benefit ratio may shift [42]. Short-term notable side effects include nausea, weight loss, and headache, and more long-term effects include mild elevations in heart rate (~3 bpm) and blood pressure (< 2 mmHG), and weight gain [43]. Notably, duloxetine does not prolong the QTc interval [44].

Duloxetine has evidence supporting its use to treat depression in children and adolescents. A network meta-analysis and comparative efficacy study of pediatric anti-depressants ranked duloxetine third behind fluoxetine and desipramine [20]. Regarding pain, only one placebo-controlled trial evaluating duloxetine for juvenile fibromyalgia exists and it demonstrated no statistically significant difference in their primary outcome measure of reduced 24-hour average pain when compared to placebo, but it did show a statistically significant increased likelihood of achieving 30% and 50% reductions in average pain. Other secondary outcomes showed improvements, such as improved activity and relationships, but these did not meet statistical significance [45]. 

#### 3.3.2. Venlafaxine

Venlafaxine has 30:1 affinity for 5-HT compared to NE making it the least noradrenergic of the SNRIs. Venlafaxine possesses sodium channel activity and, interestingly, has opioid receptor activity with one study showing a loss of its anti-depressant effects in opioid-receptor knock-out mice [16]. Venlafaxine has FDA indications for major depression, generalized anxiety, social anxiety, and panic disorder in adults, and has demonstrated efficacy in treating various neuropathic pain states, migraine prophylaxis, chronic tension type headache, and fibromyalgia [24,46,47,48]. Generally, analgesia in adults develops between 75–225 mg per day [16]. Upward titration tends to occur faster than with SSRI and TCAs. Nausea and activating symptoms (e.g., anxiety, insomnia, and restlessness) can become dose-limiting. Venlafaxine Extended-Release (ER) demonstrated superior tolerability compared to immediate release formulations, but still has the most significant discontinuation effects of the SNRIs, which can make weaning off this medication distressing to some patients [49,50].

In children, venlafaxine does not have any FDA-approved indications, but has evidence supporting its use to treat major depressive disorder and generalized anxiety disorder, as well as limited evidence to support its use to treat ADHD [20,51,52,53]. No studies have evaluated the role of venlafaxine to treat pediatric pain or headache. Side effects seem to be similar to those experienced by adults, but there is evidence supporting the FDA black box warning of suicidality associated with venlafaxine use in adolescents [20].

#### 3.3.3. Milnacipran 

Milnacipran has balanced 1:1 5-HT to NE activity. It is only FDA-approved for fibromyalgia in adults. It has a relatively short half-life that requires twice daily dosing with clinical effects occurring at 100 mg divided twice per day in adults [54]. Side effects and tolerability appear to be similar to duloxetine [55]. There are no completed trials for pediatric pain conditions, but one trial that was terminated early due to poor enrollment in the crossover component of the trial reported modest improvements in pain and quality of life during the initial open-label phase of the study [56,57].

### 3.4. Other Anti-Depressants

#### 3.4.1. Bupropion

Bupropion hydrochloride is not related chemically to other known antidepressants. It is technically in the aminoketone ketone class and is often referred to as a norepinephrine and dopamine reuptake inhibitor due to its mechanism of action. It is FDA-approved for major depression, seasonal affective disorder, and smoking cessation in adults [58]. It has no FDA-approved indications for pediatric patients, but some evidence supports its use as an off-label treatment for depression, attention deficit hyperactivity disorder, and smoking cessation in youth. Bupropion has the added benefit of being one of the few antidepressant medications not associated with weight gain or sexual dysfunction. Bupropion’s side effects tend to be activating, such as restlessness, insomnia, weight loss, and increased anxiety [59]. It is contraindicated in patients with seizure disorders, abrupt alcohol cessation, or eating disorders. 

Regarding pain, bupropion has been studied primarily for various types of neuropathic pain in adults and results are generally favorable at doses from 150–300 mg/day via the sustained-release (SR) formulation [11,60]. It has also been shown to alleviate both inflammatory and neuropathy-based pain-related behaviors and reduced morphine tolerance and dependence in rats [61,62]. There are no studies evaluating the efficacy to treat neuropathic pain in children.

#### 3.4.2. Mirtazapine

Mirtazapine also has a chemical structure unrelated to other antidepressants. Mirtazapine acts as an antagonist at the central presynaptic alpha-2-adrenergic receptors resulting in an increase in central serotonergic and noradrenergic activity. Mirtazapine also potently antagonizes 5-HT2, 5-HT3 receptors and histamine (H1) receptors, and moderately antagonizes alpha-1 adrenergic and muscarinic receptors [13]. It is FDA-approved for treatment of major depressive disorder in adults. Mirtazapine is sometimes the preferred agent for depression with associated anxiety, insomnia, and low appetite. This is because the side effects of mirtazapine include sedation, increased appetite, and weight gain. 

Regarding pain, is has been used off-label with some success for chronic tension type headache in adults [63]. Recently, it was shown to improve early satiety, nutrient intake and weight, and overall symptoms in non-anxious and non-depressed adults with functional dyspepsia [64]. This was independent of any discernable changes in gastric physiology. Mirtazapine was studied in adults with fibromyalgia without comorbid depression and shown to reduce pain and improve in quality of life [65,66]. A recent systematic review also supported mirtazapine’s role in reducing pain, and improving sleep and quality of life in adults with fibromyalgia with doses of 15–30 mg/day; however, the number needed to treat to achieve benefit was greater than those of SNRIs and pregabalin [67]. This makes mirtazapine a reasonable off-label treatment for adults with fibromyalgia who fail other treatments. Appetite stimulation and weight gain can sometimes limit treatment, so it is probably best suited for those for whom low appetite or low weight may contribute to symptoms. 

Mirtazapine has data supporting its use as an adjunctive treatment for major depressive disorder in children [20,68]. Two small case series demonstrated effectiveness and tolerability treating patients with GI symptoms and psychiatric co-morbidities including Avoidant/Restrictive Food Intake Disorder and cyclic vomiting syndrome [69,70]. Studies investigating mirtazapine’s analgesic effects in pediatric patients are currently lacking.

## 4. Alpha 2 Delta Ligands

Alpha 2 delta ligands describe the class containing gabapentin and pregabalin, and the term refers to their presumed analgesic mechanism. These agents are also referenced collectively as gabapentinoids. At the time of this review, gabapentinoids are proposed as treatments for various pain and psychiatric conditions, as well as neuropathic itch, chronic cough, restless leg syndrome and insomnia, tremor, chronic hiccups, and more Table 1 [71]. Gabapentinoids are often tried for various conditions related to excess nerve activity and as such are becoming some of the most prescribed medications worldwide. This review will limit the scope to pain and psychiatric indications. 

Gabapentin was initially developed to be a synthetic analogue of the neurotransmitter gamma aminobutyric acid (GABA); however, subsequent studies showed that it works primarily by reducing the release of excitatory neurotransmitters (such as glutamate) by the pre-synaptic inhibition of voltage-gated calcium channels by selectively antagonizing the alpha 2 delta subunit of that channel. Limited evidence also suggests effects on sodium channels and NMDA receptors, as well as possible supraspinal effects on the medial prefrontal cortex—an area of the brain associated with affective responses to pain. The alpha 2 delta-1 subunit is also important for synaptogenesis, and is upregulated by the process of central sensitization [72,73]. Gabapentin and pregabalin are mechanistically similar agents whose primary differences are pharmacokinetics. Gabapentin absorption is slower, reaches peak plasma concentrations in 3–4 hours, and has a half-life of 5–7 hours. It is absorbed through a saturable active transport system in the proximal small bowel and, with increasing doses, the bioavailability decreases from 80% with a 100 mg dose to 27% with single dose of 1600 mg [71,74]. Co-administration with magnesium salts reduces the bioavailability further. The same transporter is responsible for transport through the blood-brain barrier. Morphine increases the area under the curve of gabapentin with the proposed explanation being that intestinal hypomotility from morphine promotes gabapentin absorption [71]. Pregabalin reaches peak levels within one hour, is not absorbed through a saturable transporter, and has a bioavailability of ~90% irrespective of dose administered [75]. Both agents potentiate the central effects of opioids, a therapeutic interaction that can be both desirable and adverse depending on the context. Part of the appeal of gabapentinoids to treat various chronic conditions is that they have limited drug–drug interactions and are not hepatically metabolized. They are eliminated by the kidneys and should be dose reduced with chronic kidney disease.

The analgesia offered by gabapentin is established for various pain conditions in adults, typically with doses of 1800–3600 mg divided evenly between three doses per day. It was originally FDA-approved for post-herpetic neuralgia (PHN), but that label has expanded to include other chronic neuropathic pains after studies demonstrated efficacy in painful diabetic peripheral neuropathy (PDPN), phantom limb pain, and mixed-neuropathic states. For the most studied conditions, PHN and PDPN, meta-analysis showed a number needed to treat for benefit (NNTB) of approximately 6–7 to result in either a 30% or 50% reduction in average pain levels. For other neuropathic pain states, the results are mixed, but generally trend towards similar outcomes [76,77]. 

Gabapentin has also been studied for non-neuropathic pain conditions resulting from central sensitization, such as fibromyalgia. Despite common use, the Cochrane review found only one short-term study that met inclusion criteria for fibromyalgia [78]. That study showed a 30% reduction in pain in 49% of patients receiving gabapentin up to 2400 mg/day compared to 31% of patients receiving placebo. In the same study, 68% of patients reported pain as “better” in the gabapentin group compared to 35% in the placebo group. Gabapentin has been proposed as treatment for other central sensitization disorders, such as irritable bowel syndrome (IBS), but research is limited. One RCT of patients with diarrhea predominant IBS used short term, relatively low dose gabapentin (max 600 mg/day) to evaluate for change in symptoms (bloating, discomfort, and pain) related to experimentally induced rectal stretch and found that patients exhibited improved sensory thresholds and reduced symptoms when compared to those receiving placebo [79]. 

Pregabalin is similar to gabapentin with respect to analgesic outcomes in adults. A recent Cochrane review of its use for various neuropathic pains found dose-dependent and condition specific effects [80]. For post-herpetic neuralgia, pregabalin was much more reliable than gabapentin with a NNTB of 3.9 for 300 mg/day and 2.7 for 600 mg/day to achieve a 30% reduction in pain. The NNTB for a 50% reduction in pain were 8.3 (150 mg/day), 5.1 (300 mg/day), and 3.9 (600 mg/day), and these results were comparable to the patients’ global impression of change. In contrast, for painful diabetic neuropathy the NNTB was ~6 to achieve both 30% and 50% reduction in pain with doses of 600 mg/day. For lower doses, the NNTB was greater than 10. For mixed neuropathic pain states, the calculated NNTB was 7–8 for 30% and 50% reduction in pain, and patient global impression of change [76,80].

When evaluating pregabalin for fibromyalgia in adults, the NNTB is fairly high. To achieve a NNTB of less than 10, one must lower expectations to a 30% reduction in pain (NNTB of 7–9) or use 450 mg/day to achieve a 50% reduction in pain (NNTB of 9.7). Higher and lower doses were less effective in the analysis [81]. Patient global impression of change was also not very high and ranged from a NNTB of 7.8 (600 mg/day) to 11 (300 and 450 mg/day) to achieve the moderate benefit of “much or very much improved.” Another strategy is the combination of pregabalin and duloxetine to treat fibromyalgia. Gilron et al, performed a small 6-week, double-blind, crossover-controlled RCT of pregabalin alone, duloxetine alone, the combination of the two, and placebo [82]. For the study’s primary outcome of reduction in average pain scores, they found that combination therapy (28% reduction in average pain) was better than both pregabalin (1.4% reduction) and placebo (7.1% reduction), and that pregabalin monotherapy with a mean dose of 408 mg/day was no better than placebo. The reductions in average pain for the combination therapy group was not statistically significant from duloxetine monotherapy. Global impressions of “at least moderate global pain relief” were found in 68% receiving combination therapy, 39% with pregabalin monotherapy, 42% with duloxetine monotherapy, and 18% with placebo [30]. For irritable bowel syndrome, two studies have looked at the effects of pregabalin, both without dramatic changes in pain. However, both studies also demonstrated improvements in other uncomfortable sensations, including bloating and desire to defecate [83,84].

These agents have also been studied for painful conditions unrelated to central sensitization and nerve injury. For instance, gabapentinoids are regularly used in peri-, and post-operative settings to reduce procedural anxiety, reduce post-operative nausea and emergence agitation, reduce post-operative pain and opioid consumption. This is due to very mixed results in the literature dependent on surgical procedure and study method. A recent meta-analysis found that there was no benefit above placebo, and concluded that there was no evidence for the routine use of gabapentin in the peri-operative setting [85]. For chronic low back pain, gabapentin has not shown superiority over placebo, and pregabalin did not show superiority over the studied control analgesics (celecoxib, amitriptyline, and tramadol) [86]. Pregabalin is also used for recurrent pain conditions such as chronic pancreatitis. One industry-funded, short-term study demonstrated short-term improvements in pain and opioid reduction, but did not report medium to long-term outcomes [87]. Gabapentin was studied for prophylaxis of episodic migraine in adults and was found to be ineffective. Pregabalin has not been studied for migraine [88].

From a psychiatric perspective, gabapentinoids have been shown to exert central effects on the left anterior insula and left amygdala and attenuate their response to emotionally charged visual stimuli in healthy adults [89]. Gabapentin was found to be effective in one study to reduce social anxiety but has not been studied for generalized anxiety disorder. It may have modest effects treating substance use disorders and withdrawal symptoms [90]. Evaluations into its use to treat bipolar disorder did not show gabapentin separating from placebo. Pregabalin appears to be efficacious in treating generalized anxiety disorder with six RCTs demonstrating superiority over placebo. Furthermore, pregabalin is rapid acting with evidence in the literature of pre-procedural anxiolysis over the span hours, whereas other daily agents (namely SSRIs) take weeks to reduce anxiety. In a large placebo-controlled trial of pregabalin, venlafaxine, and placebo, pregabalin showed efficacy on day four (the first day studied), whereas venlafaxine and placebo did not.

In pediatrics, there is limited evidence to guide the use of gabapentinoids for pain or psychiatric indication. A 2016 single center, randomized, blinded study evaluated gabapentin against amitriptyline for the treatment of neuropathic pain and complex regional pain syndrome (CRPS) in children. Patients received gabapentin 300 mg three times per day or amitriptyline 10 mg at bedtime with placebo to maintain blinding. The study concluded that both gabapentin and amitriptyline were mildly effective, reducing average pain scores by 1.77 and 1.5, respectively. The authors also used a minimally important difference score of pain reduction by 1 point on the color analogue scale, and found that gabapentin was slightly more likely (60%) than low-dose amitriptyline (46.2%) to meet this threshold. This threshold is lower than the 30% reduction in pain used in other studies. One RCT evaluated the use of pregabalin for juvenile fibromyalgia in patients 12–17 years old. The pregabalin group trended towards improvement with statistically significant differences in many weeks of the study; however, by end of study, the primary outcome of average pain reduction was not statistically significant. The only improvements that were significant were global in nature with children and parents reporting “much or very much improved” in 53% of those receiving pregabalin versus 25–29% of those receiving placebo [91]. Outside of these two RCTs, the evidence supporting the use of gabapentin in children for chronic or neuropathic pains is predominately limited to case series and retrospective chart reviews, most of which find favorable results for various neuropathic or neuroirritability indications [92,93].

The potential benefits of gabapentinoids must be balanced against potential adverse effects. Overall, gabapentinoids are well tolerated. They both carry a black box warning from the FDA that they may increase suicidal thinking in adolescents. Pregabalin and gabapentin both carry concerns for abuse potential, particularly in those already with a substance use disorder. In adults, pregabalin was found to have euphoric and sedative properties similar to other abused substances, and also demonstrated tolerance, physical dependence and withdrawal phenomena [94]. This resulted in pregabalin being categorized as a Schedule V (abuse potential) in the USA. This has not been studied in pediatrics. Other common side effects of these agents include dizziness, headache, nausea, sedation, and weight gain.

## 5. Mood Stabilizers

This class of medications utilizes multiple mechanisms of actions, but generally overlaps with anti-epileptic medications. Despite the overlapping psychiatric and neurological uses, this class contains significant in-class variability. As such, most of these agents will be addressed separately.

### 5.1. Oxcarbazepine and Carbamazepine

Oxcarbazepine and Carbamazepine are agents that share a similar mechanism of action, though they are metabolized differently, and due to these similarities will be reviewed together. Carbamazepine was initially approved in 1968, and is FDA-approved for trigeminal neuralgia, generalized tonic-clonic seizures, and partial, complex seizures in adults. Oxcarbazepine is newer, does not have any FDA-approved indications for neuropathic pain, and is approved to treat partial seizures in adults and children down to 2 years of age. Both agents bind to the alpha sub-unit of the voltage-gated sodium channel (Nav) in the inactive state. Advances in electrophysiology and pharmacogenomics have led to the characterization of sodium polymorphisms associated with increased risk of chronic pain in conditions such as adult osteoarthritis, fibromyalgia, and post-surgical pains, as well as pathogenic mutations in SCN9A (encoding Nav1.7) that underlie cases of inherited erythromelalgia and extreme pain disorder [95,96,97]. These advances have led to the renewed interest in sodium channels antagonists to treat pain. 

Clinically, carbamazepine is FDA-approved to treat trigeminal neuralgia in adults, and has evidence supporting its use to treat diabetic neuropathy, glossopharyngeal neuralgia, and phantom limb pain. Oxcarbazepine demonstrated similar efficacy for trigeminal neuralgia and diabetic neuropathy, as well as mixed-cause polyneuropathy and central pain related to multiple sclerosis [98]. Demant et al. proposed that a pain phenotype known as the “irritable nociceptor” (IN) may be particularly susceptible to oxcarbazepine [99]. In this phenotype, patients describe neuropathic pain with preserved thermal and pinprick (i.e., small fiber) sensations and evidence of “gain of function” (e.g., increased pain or a heightened sensation of cold) with Quantitative Sensory Testing (QST). In a prospective, randomized, placebo-controlled, phenotype-stratified study, the authors found that 29% of patients achieved 50% reduction in pain with the IN phenotype, whereas there was minimal improvement in those with the non-irritable phenotype. Improvements were also seen in deep, aching pain, pain evoked by light pressure, lancinating pain, and a there was a greater reduction in sleep-related pains in those patients with IN pain treated with oxcarbazepine. This led to an estimated number needed to treat for benefit (NNTB) of 3.9 for the IN phenotype, and a NNTB of 13 for the non-irritable phenotype. A later Cochrane review that did not account for phenotype estimated an overall oxcarbazepine effect of NNTB of 6 for treatment of neuropathic pains [100]. The interventions in these trials generally started with 300 mg per day and titrated to clinical effect over 3–4 weeks with an average effective dose ranging from 600–1200 mg divided twice per day and a typical max of 1800 mg divided twice per day [98].

In the field of psychiatry, carbamazepine is FDA-approved for the treatment of acute mania and mixed episodes associated with bipolar disorder type 1 in adults. In children, there are scarce case reports that the medication is effective in treating youths with bipolar-1 disorder, and several open-label studies evaluating its use for pediatric ADHD [101,102]. Oxcarbazepine does not have any FDA-approved psychiatric indications, but some studies in adults have demonstrated non-inferiority compared to carbamazepine, and less drug–drug interactions and side effects. This suggests that oxcarbazepine may be preferred over carbamazepine, despite being off-label, when conventional treatments have failed [103].

Despite having a similar mechanism of action, side effects and drug-interactions of oxcarbazepine and carbamazepine are distinct due to differences in drug metabolism and with a clear superiority of oxcarbazepine in this regard. Carbamazepine is metabolized by the cytochrome p450 pathway, is prone to multiple drug interactions, and induces its own metabolism. Oxcarbazepine has only minor interactions with CYP3A4 with doses up to 900 mg/day, and its primary pathway of metabolism via CYP2C19 is not shared with many other agents making it well-utilized in complex patients with polypharmacy, including psychotropic agents. Carbamazepine is associated with the serious adverse effect of agranulocytosis while oxcarbazepine is uniquely associated with syndrome of inappropriate antidiuretic hormone (SIADH) and hyponatremia. The causes underlying these differences are not fully understood. Both agents can lead to Stevens–Johnson Syndrome (SJS), particularly in those with increased genetic risk such people of Asian descent, and it is recommended that patients at risk be tested for HLA-B*1502 and HLA-A*31:01 haplotype before initiating therapy due to a 10-fold increased risk of SJS. Other recently suggested adverse effects include an increased rate of neurodevelopmental disorders (primarily Autism Spectrum Disorder) to children born of women taking oxcarbazepine during pregnancy or breastfeeding, an average 1 cm/year reduced linear growth velocity of children with epilepsy taking oxcarbazepine [104,105]. Other common side effects include sedation, headache, weight gain, dizziness, double vision, vertigo, and agitation.

No studies have evaluated the efficacy of oxcarbazepine or carbamazepine to treat pain in children, and only one case report could be found that documented improvements in a child with CRPS refractory to gabapentin. 

### 5.2. Lamotrigine

Lamotrigine does not have conclusive empirical support nor FDA-approved indications to treat psychiatric conditions in children or adolescents. Much of the data exploring lamotrigine for bipolar disordered youth is retrospective or in open-label trials. Despite this, lamotrigine is occasionally used off-label to treat pediatric mood disorders [106]. Lamotrigine has been evaluated by multiple studies for various pain states due to its multiple mechanisms of action including sodium-channel inhibition, presynaptic neuronal stabilization, and central glutamate suppression. Pre-clinical work suggested anti-hyperalgesic effects in various neuropathic pain models [107,108]. Interestingly, one placebo-controlled study evaluated the effect of a pre-surgical lamotrigine load, similar to common practices with gabapentin, and found an anti-hyperalgesic effect when compared to both placebo and topiramate with outcomes of reduced analgesic rescues in the post-anesthesia care unit (PACU) and shorter PACU stays [109]. Despite those possible effects, lamotrigine is largely ineffective at treating chronic or neuropathic pains in adults, outside of neuropathic pain related to incomplete spinal cord injury, human immunodeficiency virus (HIV) neuropathy, and migraines with complex aura (e.g., migraine with brainstem aura or central vertigo) [110,111,112]. It may be modestly effective for trigeminal neuralgia, but with less tolerability than typical alternatives [113]. It is commonly associated with rash, most notably Stevens–Johnson Syndrome, but it is also associated with other drug related rashes such as Drug Reaction with Eosinophilia and Systemic Symptoms (DRESS) syndrome [114]. Lamotrigine does not have any specific evidence supporting its use for pediatric pain conditions.

### 5.3. Topiramate

Topiramate is an anti-epileptic that does not have any FDA-approved psychiatric indications in adults or children. It is sometimes used to treat mood instability, particularly in patients with co-morbid headache, but the evidence supporting this practice is equivocal. A recent Cochrane Review could not draw any firm conclusions about the use of topiramate in treating bipolar disorder in adults, and showed moderate-quality evidence that topiramate was no more effective than placebo [115]. Similarly, an initial open-label trial of topiramate to treat PTSD showed promise; however, subsequent metanalysis did not show a benefit above placebo [116]. Outside of psychiatry, topiramate is FDA-approved for various epilepsy syndromes and for migraine prophylaxis in adults and children over age 12 [117]. Its mechanism of action is thought to be related to inhibition of voltage-gated sodium channels, reducing alpha-amino-3-hydroxy-5-methyl-4-isoxazolepropionate (AMPA)/Kainate stimulated membrane destabilization, enhancing GABA A receptor activity, and as a weak carbonic anhydrase inhibitor [118]. 

Due to its multiple effects on pain-related neural transmission, it has been studied in neuropathic states in adults without great success [119]. Several small trials showed improvements with lumbar radiculopathy, and some possible efficacy in phantom limb pain, though the doses used to achieve effects were extremely high (mean 800 mg/day) [119,120]. Topiramate does have efficacy in adult migraine, particularly in studies of chronic migraine and chronic daily headache [121,122]. This may be related to its multiple effects and mechanisms of action, including its anti-obesity effects and carbonic anhydrase inhibition. For these reasons, it sometimes used as an adjunctive medication for the treatment of idiopathic intracranial hypertension [123,124]. It also has efficacy in treating trigeminal neuralgia and trigeminal autonomic cephalalgias [125]. Side effects are common, and are typically experienced as brain fog, appetite suppression, paresthesia, and renal stones. It has teratogenic potential, particularly when used with other therapies [126]. With dosing over 200 mg/day, topiramate can reduce the effectiveness of oral contraceptive therapies. In female patients of childbearing age, folate supplementation is recommended, particularly in those with contraceptive forms that are less reliable in the adolescent population [37].

Despite its FDA-approval for pediatric migraine, a recent multi-center double blind, cross-over controlled trial (CHAMP) cast doubt on its effects for migraine prevention beyond that of placebo [35,36]. Furthermore, network meta-analysis supported these findings. The American Academy of Neurology and American Headache Society 2019 practice guideline for pharmacologic prophylaxis of pediatric migraine concluded that topiramate probably reduces migraine days when compared to placebo, but that currently available data do not support topiramate’s ability to achieve typical research outcomes of reducing migraine frequency by 50%, and is unlikely to reduce migraine-related disability (as measured by the Pediatric Migraine Disability Assessment-PedMIDAS) when compared to placebo [37].

### 5.4. Lithium Carbonate

Lithium carbonate is a highly soluble salt that is primarily excreted by the kidneys. It is FDA-approved for the treatment of bipolar mania and maintenance therapy of manic-depressive patients as young as age 12 [127]. This approval came mainly from studies conducted in adults. The mechanism by which lithium exerts its mood stabilizing properties is not well understood. One of the major difficulties in prescribing this medication is its low therapeutic index. Toxicity may occur at serum levels just slightly above those necessary to achieve therapeutic effects [128]. Toxicity may manifest as diarrhea, vomiting, ataxia, tremor, sedation, and slurred speech. Severe toxicity or overdose can lead to cardiac arrythmias, seizures, stupor, coma, or death. Lithium requires routine lab monitoring to assess serum levels, as well as to monitor kidney function and thyroid function, which can become affected by lithium use. In general, lithium should not be administered in people who are pregnant (or likely to become pregnant) due to a small but significant risk of cardiac teratogenicity. 

Lithium has not been evaluated in many human trials as a treatment for pain outside of cluster headaches and in neuropathic pain related to spinal cord injury [129,130,131]. There is a small amount of data in rat pain models suggesting that lithium is anti-allodynic, has visceral anti-hyperalgesia properties, and may reduce paclitaxel induced neuropathy [132]. There are no studies evaluating the efficacy of lithium to treat pain in children.

### 5.5. Sodium Valproate

Sodium valproate is an anti-epileptic agent used in nearly all seizure types. It is FDA-approved for treatment of acute mania associated with bipolar disorder in adults but has no FDA-approved psychiatric indication for children and adolescents. Its mechanism of action is related to voltage-gated sodium channel inhibition, augmentation of GABA, and mild inhibition of T-type calcium channels. Sodium valproate has been evaluated for numerous neuropathic pains in adults, and has some evidence suggesting efficacy treating post-herpetic neuralgia and diabetic neuropathy [133]. Outside of post-herpetic neuralgia and diabetic neuropathy, there is no evidence for use in other pains states. The data that exist for those conditions are limited, and there are numerous concerning side effects with valproate including teratogenicity, pancreatitis, SIADH, hyperammonemia, and cerebral edema. It is sometimes used as an intravenous treatment for status migrainosus in adults and children [134,135]. Treatment with oral forms of the drug is still recommended for several primary headache disorders, but the therapy has generally fallen from favor due its side effects and unfavorable risk-benefit ratio, particularly in females of childbearing age.

The safety and efficacy of this medication in pediatric psychiatry remains to be fully understood. Notable studies show a lack of benefit over placebo when treating pediatric bipolar disorder or show less efficacy when compared with other agents [136,137]. There were no identified studies evaluating valproate for pediatric pain conditions outside of its well supported but challenging use as a migraine therapy.

## 6. Antipsychotics

The use of antipsychotic medications in patients with pain is a relatively new concept considering that older, first generation antipsychotics are notorious for their unwanted effects such as extrapyramidal symptoms and dystonic reactions [138]. Now that second generation (atypical) antipsychotics, are more widely used and available, the use of antipsychotics in the treatment of patients with pain symptoms is being revisited for treatment of pain and headache in adults. This is because of their multiple mechanisms of action and evidence of efficacy for treating many co-morbidities associated with pain including insomnia, refractory depression, and anxiety [139,140,141,142]. Atypical anti-psychotics affect many receptors that may modulate pain including 5-HT receptor agonism and antihistaminic, anti-alpha adrenergic, and anticholinergic activity. 

In a recent systematic review of atypical antipsychotics for the treatment of adult chronic pain syndromes, olanzapine was found to demonstrate preliminary efficacy to treat fibromyalgia, chronic headache, and central sensitization [138]. Other agents were evaluated but failed to demonstrate efficacy in the reviewed studies. A different review, as part of the Cochrane Library, evaluated the use of antipsychotics to treat fibromyalgia and concluded that quetiapine provided mild analgesia in some people with a NNTB of 8 to achieve a 30% reduction in pain using doses of 50–300 mg per day [141]. There was significantly improved sleep, depression, and anxiety, as well as a higher retention rate in the study group when compared to placebo. A comparative evaluation of quetiapine and amitriptyline suggested similarities between the non-analgesic effects of quetiapine and amitriptyline; however, there were more side effects associated with quetiapine therapy [143]. The authors suggested that quetiapine may have a role as a short-term therapy for many of the co-morbidities associated with fibromyalgia, even if the analgesic benefits are limited.

No pediatric studies for complex pain or headache conditions were found in the literature. One pediatric retrospective study found benefit for acute headache or abdominal pain in an emergency room setting, but extrapolation to other uses in this population is limited [144].

Side effects of atypical antipsychotics must be considered in treatment planning for patients with comorbid psychiatric and pain diagnoses (Table 2). In addition to extra-pyramidal side effects, weight gain, and dyslipidemia, sedation and hypotension can also be seen in atypical antipsychotics. The secondary effect of weight gain is important to consider, as this can become counterproductive to treatment and contribute to further functional decline.

## 7. Anti-Sympathetic Agents

Anti-sympathetic agents are not commonly utilized as psychotropic medications, but they do seem to have a role in patients experiencing symptoms attributed to excessive sympathetic nervous system activation (Table 3). A classic example is the use of propranolol for performance anxiety, but it has evolved to include other uses such as for panic disorder, generalized anxiety, and PTSD. It is also commonly used in conditions of hyperadrenergic excess such as Postural Orthostatic Tachycardia Syndrome (POTS). The alpha-2 agonists clonidine and guanfacine are FDA-approved for ADHD and tic disorder related to Tourette’s Syndrome, but are also used in PTSD, Oppositional Defiant Disorder (ODD), excoriation disorder, and insomnia [145]. Furthermore, evolving basic science research suggests an analgesic action to certain anti-sympathetic agents. 

### 7.1. Beta-Blockers

Beta-blockers constitute a class of agents that are classified as non-selective or selective beta adrenoreceptor antagonists. This class is not normally included as a psychotropic agent. However, it retains use in psychiatry for anxiety disorders, particularly performance anxiety and in blunting misappraisals of bodily sensations (e.g., orthostatic symptoms) that can lead to panic reactions [146]. For those reasons, and the use of these agents treating pain in disorders attributed to the sympathetic nervous system, such as Postural Orthostatic Tachycardia Syndrome (POTS), this class was included for review.

Over the past decade, several studies examined the role of beta adrenoreceptors in various experimental pain states in rats and humans with mixed results. For example, multiple mechanistic rat studies demonstrated that beta-2 receptor agonist activity is important for the treatment of allodynia using the anti-depressants nortriptyline and duloxetine with suggestion that some of the effects of the descending inhibitory pain system are mediated by activation of the beta-2 receptor [147,148].

Other recent studies have demonstrated that sustained stimulation of the beta-2 and beta-3 receptors can lead to persistent functional pains and neuroinflammation in the rat, and that this persistent activation may be related to haplotypes associated with low activity of catechol-O-methyltransferase (COMT) [149,150]. In rat and human studies that select for low COMT activity haplotypes, non-selective beta blockers, namely propranolol, showed reductions in chronic musculoskeletal pains and pain related to temporomandibular disorder. The improvements appeared to be mediated by the peripheral antagonism of the beta-2 receptor [149,151]. Interestingly, in rats with experimentally induced low COMT activity, propranolol had a marked reduction in pain sensation and the affective component of pain as manifested by pain anxiety-related behaviors [151]. No conclusions or specific dose recommendations can be made at this time, but due to the documented co-prevalence of anxiety and pain disorders, particularly in patients with low COMT activity, further research is warranted.

In psychiatry, propranolol is often used for performance anxiety. Its use as a front-line anxiolytic therapy has diminished over time, but it retains research interest in the use around fear and memory, particularly around PTSD. A systematic review of propranolol found no evidence that it separated from placebo in the treatment of PTSD; however, studies comparing it to benzodiazepines found it to be equivalent and without the unwanted adverse effects of benzodiazepines [146]. The review further suggested that propranolol may have a role in breaking the cycle of catastrophic misappraisal of bodily sensations of orthostatic origin that can contribute to panic attacks. 

Clinically, no studies have evaluated beta-blockers to treat chronic pain in children. It does have a long history of use for prevention of migraine. A recent network meta-analysis found that propranolol may have short term improvements for pediatric migraine comparable to topiramate, but that effects appeared to diminish over time [36].

### 7.2. Alpha-2 Agonists

Alpha-2 agonists, namely clonidine, are frequently used by pediatric pain specialists to enhance the effectiveness of opioids, to serve as an adjunctive therapy for neuropathic and cancer pain, reduce symptoms of opioid withdrawal, and to provide sedation [152,153,154]. The proposed mechanism of these effects is via pre-synaptic spinal interneuron inhibition of ascending pain transmission. Clonidine is often the agent of choice due to its wide range of administration routes, low cost, and long history of use in pediatric anesthesia. Dexmedetomidine is also accumulating evidence of efficacy in similar conditions, that further suggests a class mechanistic effect and reinforces the role of the alpha-2 adrenoreceptor in reducing pain transmission [155]. Guanfacine has limited pre-clinical work demonstrating similar efficacy, but little clinical research or experience.

Alpha-2 agonists also are FDA-approved for use in children for the treatment of ADHD and management of tics associated with Tourette Syndrome. It is sometimes used off-label for treatment of excoriation disorder, oppositional defiant disorder, insomnia, and PTSD. For these indications, these agents appear to reduce norepinephrine levels in the pre-frontal cortex, leading to improved impulsivity and hyperactivity [145]. Clonidine is generally well tolerated, and its side effects are expected from its mechanism of action, including dry mouth, hypotension, urinary retention, constipation, and weight gain. For the treatment of pain in children, no clear dosing recommendations are available, though previous recommendations include a starting doses of 1–3 mcg/kg/dose at bedtime (typical max initial dose of 0.1 mg) and increasing the interval to every six hours as tolerated [152].

## 8. Stimulants

There is scant literature on the overlapping comorbidity of children with ADHD and chronic pain. However, some studies suggest that rates may be as high as 18–19.9% in children with chronic pain [7,156]. Furthermore, a study investigating comorbidities in pediatric headache found that 28% of children who had been diagnosed with a primary headache disorder had also been diagnosed with ADHD [157]. These results are significant considering that the prevalence for ADHD in the general child population is 9.4%, and imply that careful consideration should be given to how children with chronic pain and headaches respond to medications for ADHD [158].

Very limited evidence suggests stimulants may provide some analgesic utility. In one experimental pain study of adult volunteers with ADHD, methylphenidate prolonged the ability of subjects to tolerate cold pain that was independent of other non-painful aversive stimuli [159]. Additional pre-clinical work in rats suggests that low doses of stimulants may enhance the analgesic properties of opioids and provide an opioid sparing effect [160]. Finally, while not directly related to analgesic properties, stimulants may have an important role at reducing cognitive fatigue and improving alertness in patients with life-limiting illness and chronic opioid therapy.

## 9. Conclusions

Psychotropic medications are a heterogenous group of medications with multiple mechanisms of action, some of which may reduce pain or other frequently associated symptoms such as nausea, insomnia, and fatigue. These effects seem to be independent of their psychiatric effects. Little is known of the efficacy of these agents to reduce pain in children and adolescents. There is a greater understanding of the role of these medications for psychiatric indications. Investigating psychiatric or developmental co-morbidities in children and adolescents with chronic pain and headache may help improve chances for therapeutic success, as well as helping patients globally with their ability to attend school and participate in their usual activities. For complex patients with both challenging pain and psychiatric co-morbidities, careful attention to medication selection may provide patients with multiple avenues to improvement.

## Figures and Tables

**Table 1:** Agents that have evidence to support use for psychiatric, pain and related symptoms.

Medication	Anxiety/Depression	ADHD	Bipolar Disorder	Insomnia	Anorexia	Schizophrenia	Migraine	Neuropathic Pain	Fibromyalgia	Comments
Antidepressants										
SSRIs	✓✓				✓✓					1st line medication for pediatric depression and anxiety
TCAs	✓✓	✓✓		✓✓			✓	✓	✓	Low doses used for pain often insufficient to improve mood
SNRIs	✓✓	✓✓ (limited)					✓ (venlafaxine)	✓	✓	Can be considered 1st line for depression and anxiety with comorbid pain syndrome
Bupropion	✓✓	✓✓						✓		Not associated with weight gain. Can assist in smoking cessation
Mirtazapine	✓✓			✓	✓✓				✓	Associated with sedation and appetite increase.
Mood stabilizers										
Lamotrigine			✓				✓ (complex aura)	✓ (limited)		
Topiramate				✓			✓✓			
Lithium			✓✓							Renally excreted
Oxcarbazepine			✓✓ (limited)							
Carbamazepine			✓✓ (limited)							Many drug–drug interactions
Valproic Acid			✓✓				✓	✓		Teratogenic
Antipsychotics										
Olanzapine	✓		✓✓	✓	✓	✓✓			✓	Can be helpful for acute agitation
Quetiapine	✓		✓✓	✓	✓	✓✓			✓	
Others	✓		✓✓	✓	✓	✓✓				Risperidone or aripiprazole have evidence to support its use for irritability associated with autism
Anti-Sympathetics										
Alpha-2 agonists	✓	✓✓		✓✓				✓✓		
Beta-blockers	✓ (somatic anxiety)						✓✓			

✓✓ = Evidence supporting use in pediatrics; ✓ = Evidence supporting use in adults

**Table 2:** Comparison of side effects of psychotropic agents and authors’ estimates of clinical impact.

Medication	Restlessness/Activation	Insomnia	GI Distress	Headache	Weight Gain	Sedation	Hypotension	Suicidality	QTc Prolongation	Comments
Antidepressants										
SSRIs	+	+	++	+	+	+		+	+ (citalopram most clinically significant)	Generally safe. GI and headache side effects usually transient.
TCAs			++ (constipation)		++	+	+	+	+	Significant cardiac risk if used in an overdose.
SNRIs	+	+	++	+	+			+	+ (venlafaxine)	May be more difficult to reduce or discontinue due to discontinuation effects.
Bupropion	++	++						+	+	Contraindicated in eating disorder and epileptic patients.
Mirtazapine					++	++	+	+	+	Weight and sedation effects can be used to the prescriber’s advantage in patients with insomnia and/or poor PO intake.
Mood stabilizers										
Lamotrigine		+	+	+		+		+		Risk of rash or Stevens–Johnson Syndrome (SJS). Slow titration required to avoid SJS.
Topiramate	+					+		+		Brain fog, paresthesias, renal stones, teratogenicity.
Lithium			+		++	++				Low therapeutic index. Fetal risk.
Carbamazepine			+			+				Risk of SJS and agranulocytosis.
Oxcarbazepine			+			+				Better side effect profile and less drug–drug interactions when compared to carbamazepine. Clinically significant hyponatremia.
Valproic Acid					++	++				Fetal risk, pancreatitis, and hepatotoxicity
Antipsychotics										
Olanzapine			+	+	+++	++	+			
Quetiapine			+	+	++	++	++		+ to ++	
Others			+	+	++	++	+ or ++		+ to +++	
Anti-Sympathetics										
Alpha-2 agonists			+	+	+	++	++			
Beta-blockers					+	+	++			Dose dependent effects on orthostatic dizziness

+ = mild effects, ++ = moderate effects, +++ = major possible effects.

**Table 3:** Clinical Vignettes and Guidelines for Treatment.

Case Description	Medications to Consider	Target Symptoms
Hermione is a 14-year-old girl with a history of chronic abdominal pain and nausea related to functional dyspepsia leading to weight loss, tension-type, and associated amotivation, anhedonia, and sleep disruption	SSRI	Chronic abdominal pain, low mood, anorexia
	Mirtazapine	Weight loss, tension headaches, abdominal pain, insomnia, low mood
	Olanzapine	Weight loss, nausea, insomnia, refractory depression
Harry is a 16-year-old male with neuropathic pain from incomplete spinal cord injury resulting from suicide attempt, injury-associated PTSD, nightmares, insomnia, generalized anxiety, depression, and migraines	SNRI	Anxiety, PTSD, neuropathic pain, migraine
	Gabapentinoids	Neuropathic pain, insomnia, generalized anxiety
	Alpha-2 agonist	Insomnia, neuropathic pain, anxiety/PTSD
	Oxcarbazepine	Neuropathic pain, mood stabilization
	Lamotrigine	
Luna is a 15-year-old female with chronic migraine, panic disorder, ADHD, Postural Orthostatic Tachycardia Syndrome (POTS), and insomnia	SNRI (venlafaxine)	Migraine, anxiety, ADHD
	Alpha-2 agonist	Insomnia, ADHD, anxiety, POTS
	Low dose beta blocker	Migraine, anxiety, POTS
